# Ubiquitination of secretory granules promotes their crinophagic degradation in *Drosophila*

**DOI:** 10.1002/1873-3468.70376

**Published:** 2026-05-31

**Authors:** Tamás Csizmadia, Anna Dósa, Asha Kiran Maddali, András Jipa, Hajnalka Laczkó‐Dobos, Péter Lőw, Gábor Juhász

**Affiliations:** ^1^ Department of Anatomy, Cell and Developmental Biology Eötvös Loránd University Budapest Hungary; ^2^ Research Institute of Molecular Pathology (IMP) Vienna BioCenter PhD Program Vienna Austria; ^3^ Heidelberg University Biochemistry Center (BZH) Heidelberg Germany; ^4^ Institute of Genetics HUN‐REN Biological Research Centre Szeged Szeged Hungary

**Keywords:** Cnot4, crinophagy, glue granule, K63‐linked polyubiquitin, lysosome, quality control

## Abstract

Gland cells dynamically regulate their secretory granule content via balancing synthesis, maturation, secretion, and lysosomal degradation (crinophagy). However, the signal(s) leading to crinophagic breakdown of secretory granules are unknown. Here, we show that ubiquitination of unreleased or low‐grade glue‐containing secretory granules marks these vesicles for crinophagy in larval salivary gland cells of *Drosophila*. We identify the ubiquitin ligase Cnot4 as a key mediator of glue granule ubiquitination. Loss of *Cnot4* prevents ubiquitination and impairs granule fusion with lysosomes. Overexpression of Cnot4 induces premature crinophagy via ectopic ubiquitination of granules. Our work establishes that Cnot4‐dependent ubiquitination of secretory granules is a key trigger of crinophagy in *Drosophila*, paving the way for further analysis of this barely characterized degradation route in metazoans.

## Graphical abstract

Ubiquitination of secretory granules in *Drosophila* larval salivary glands is a critical molecular trigger for crinophagy, the lysosomal degradation of unreleased, or low‐quality granules. The E3 ubiquitin ligase Cnot4 is recruited to the surface of secretory granules to induce crinophagy. Accordingly, Cnot4 depletion impairs granule–lysosome fusion, and its overexpression induces premature crinophagy via changes in the ubiquitination status of granules.

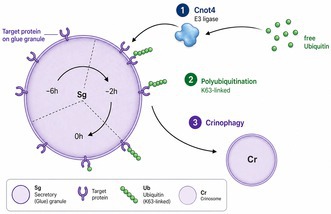

## Abbreviations

**Atg**, Autophagy related

**BDSC**, Bloomington *Drosophila* Stock Center

**CCR4‐NOT**, Carbon Catabolite Repression 4 ‐ Negative On TATA‐less

**Cnot4**, CCR4‐NOT transcription complex, subunit 4

**CORVET**, class C cORe Vacuole/Endosome Tethering

**CP**, Cytoplasm

**Cr**, Crinosome

***dor***, deep orange

**DsRed**, Discosoma (a coral genus) red

**DUB**, DeUBiquitinase or DeUBiquitinating enzyme

**E3 enzyme**, ubiquitin ligase

**Gg**, Glue granule

**HOPS**, HOmotypic fusion and Protein Sorting

**K63**, Lysine 63

**Lamp1**, Lysosome Associated Membrane Protein‐1

**LE**, Late Endosome

***lt***, light

**Lys**, Lysosome

**MVB**, multivesicular body

***Not1***, Negative On TATA‐less

***Rcd‐1*** Required for cell differentiation 1

**RING**, Really Interesting New Gene

**RNAi**, RiboNucleic Acid interference

**RPF**, Relative to Puparium Formation

**Sg**, Secretory granule

**Sgs3**, Salivary gland secretion 3

**SNARE**, Soluble NSF Attachment Protein Receptor

**Syx13**, Syntaxin 13

**TGN**, Trans‐Golgi Network

***UAS***, Upstream Activating Sequence

**Ub**, Ubiquitin

**VDRC**, Vienna *Drosophila* Resource Center

**Vid**, Vacuolar import and degradation

**Vps16A**, Vacuolar protein sorting 16A

Secretory mechanisms are essential components of numerous fundamental biological processes, including digestion, respiration, and neuronal and endocrine functions in all animals and humans. Secretory granules serve as storage organelles for secretory materials, and these undergo complex rearrangements, including growth through homotypic fusion, maturation via moderate acidification, and fusion with elements of the endo‐lysosomal system [1, 2, 3]. The maturation process alters the composition and pH of the secretory cargo to prevent premature secretion or degradation of secretory granules, implying that crinophagy functions as a secretory granule quality control mechanism [3, 4, 5, 6, 7]. Consequently, mature secretory granules are prepared for releasing their contents through exocytosis. Effective release of secretory material necessitates the coordinated recruitment and function of a secretion‐specific acto‐myosin system on the cytosolic surface of the secretory granule membrane after forming a fusion pore via merging with the plasma membrane, as observed in the *Drosophila* larval salivary glands and in the rat exocrine pancreas cells [8, 9].

The limited secretory activity of cells can result in the accumulation of unreleased secretory granules within the cytoplasm, which are frequently degraded through crinophagy. This process, although less well‐known, represents an unconventional autophagic mechanism that occurs in all secretory tissues, including exocrine, endocrine, and neuroendocrine cells [10]. Crinophagy refers to the direct fusion of superfluous secretory granules with late endosomes and lysosomes. This process results in intense acidification, digestion, and recycling of unused secretory material [10, 11, 12]. Consequently, unreleased granules transform into crinosomes, specialized secondary lysosomal compartments where the secretory contents are loosened, degraded, and recycled [11, 13]. Although the initial description of crinophagy via electron microscopic examination by Smith and Farquhar dates back to 1966 [12], the molecular mechanisms and genetic regulation underlying this process are still just beginning to unfold.

Early ultrastructural investigations in the late larval salivary gland of *Drosophila pseudoobscura* identified crinophagic degradation of mucopolysaccharide (glue)‐containing secretory granules [14]. We have shown that the fusion of glue granules with late endosomes or lysosomes in the late larval and prepupal salivary glands of *Drosophila melanogaster* is a developmentally programmed process. As a result, this organ provides a robust experimental system for investigating the intricate molecular mechanisms and regulatory pathways that control crinophagy [7, 11]. In our previous papers, we developed and extensively characterized fluorescent reporter systems to monitor the fusion of secretory granules with late endosomes and lysosomes and identified the key components involved in secretory granule–lysosome fusion within larval salivary gland cells of *Drosophila* [11, 15, 16]. However, the molecular signal(s) that trigger the selective crinophagic degradation of obsolete or low‐quality secretory granules instead of their exocytosis are still unknown.

The human body also contains several protein‐secreting gland tissues, all of which utilize the process of crinophagy to regulate their secretory granule pool. This mechanism eliminates unnecessary secretory vesicles and ensures quality control of the secretory material [7, 10, 12, 17, 18]. In the exocrine pancreas, the extensive fusion of trypsinogen granules with lysosomes may trigger the premature intracellular activation of trypsinogen by lysosomal hydrolases, such as cathepsins, leading to necrotic cell death of the gland cells and severe inflammation of the pancreas [19, 20]. Crinophagy is also believed to modulate the amount and production of insulin in the β cells of the Langerhans islets [17, 21, 22, 23]. Thus, a deeper understanding of the genetic network governing the process of crinophagy will be crucial for improving our knowledge and may be useful for potential prevention of acute pancreatitis and insulin production defects observed in type 2 diabetes.

Ubiquitin is a highly conserved protein and serves as a post‐translational modifier, playing diverse roles in cellular processes. This small molecule can be covalently conjugated to diverse target biomolecules, including proteins, lipids, and carbohydrates, in the form of mono‐, multi‐, or polyubiquitin chains that employ various linkage methods, such as Lysine‐48 (K48) and Lysine‐63 (K63) types [24, 25, 26]. Eukaryotic cells exhibit several examples of ubiquitination and selective macroautophagic degradation of damaged or obsolete cellular organelles, such as mitophagy of mitochondria, lysophagy of lysosomes, and secretophagy of secretory granules, all of which rely on capture of cargo into forming autophagosomes [7, 20, 27]. Components of the ubiquitin‐proteasome system, including E3 enzymes or deubiquitinating enzymes (DUBs), have been implied in the regulation of vesicular trafficking and autophagic pathways [24, 28, 29]. Intriguingly, in yeast cells, the vacuolar import and degradation (Vid) pathway functions as a specialized, autophagosome‐independent autophagic process akin to crinophagy, and this mechanism involves the ubiquitin ligase encoding gene *Vid24/YBR105C* [11, 30].

In this study, we examined the exciting question of how secretory granules that are unnecessary or of low quality are directed to the lysosomal compartment. We discovered that ubiquitin serves as a molecular signal on the surface of glue‐containing secretory granules when crinophagy is developmentally activated, guiding them for degradation in the late larval salivary gland of *Drosophila melanogaster*. Additionally, we conducted a genetic screen of *Drosophila* E3 enzymes (Table S1) and we identified a novel regulator of the crinophagic process: the ubiquitin ligase Cnot4, which we find to be required for ubiquitination of glue granules during normal development. Strikingly, knockdowns of *Cnot4* resulted in defects in glue granule acidification and granule‐to‐lysosome fusion, similar to previously identified essential crinophagic factors, such as *Vps16A*, *Rab7*, and *Syx13* [11]. Vice versa, overexpression of Cnot4 induced premature ubiquitination of glue granules and early crinophagy. Our findings identify ubiquitination as a fundamental molecular signal that triggers crinophagy and open a new avenue for functional analysis of this process in animals and humans.

## Materials and methods

### Fly stocks and work

The following fly stocks were obtained from the Bloomington *Drosophila* Stock Center: *Sgs3 (Glue)‐GFP (5884)* [31], *UAS‐LifeAct‐Ruby (35545)*, *UAS‐Rab6* ^*JF02640*^ *(27490), UAS‐Not1* ^*JF03096*^ and *UAS‐Rcd‐1* ^*HMS05850*^. Fly stock RNAi line obtained from the Vienna *Drosophila* Resource Centre was: *UAS‐Cnot4* ^*GD4410*^ *(v10850—Cnot4* ^*RNAi I*.^ *)*. Fly stock RNAi line obtained from NIG‐Fly was: *UAS‐Cnot4* ^*31716R‐1*^ *(Cnot4* ^*RNAi II*.^ *)*. Additional fly lines included *UAS‐GFP‐Ub* (provided by P. Deák, Department of Genetics, University of Szeged, Szeged, Hungary) [32], *UAS‐GFP‐Lamp1* and *UAS‐Vps16A* ^*RNAi*^ (provided by H. Krämer, Center for Basic Neuroscience, UT Southwestern Medical Center) [33], *Sgs3 (Glue)‐DsRed* (Glue‐Red, provided by A. Andres, University of Nevada, Las Vegas, NV) [34], and *fkh‐Gal4* [3, 11], *UAS‐Cnot4* (kindly provided by Mika Rämet, Tampere University, Faculty of Medicine and Health Technology, Finland) [35].

### Flybase dataset

RNA‐seq expression data for CG31716 were obtained from publicly available modENCODE datasets. Salivary gland samples from third instar larvae (L3; SRX029402, SRX029403) and white prepupal stage (WPP; SRX029409, SRX042031) were analyzed. All datasets are part of the modENCODE developmental time‐course project (BioProject PRJNA75285; SRA SRP001065) and were accessed via FlyBase. Expression values are based on normalized RNA‐seq read counts (RPKM) as provided by FlyBase [36, 37, 38].

### Fluorescent microscopy and immunocytochemistry

Salivary glands were dissected from control, mutant, and RNAi animals at the indicated developmental stages, fixed for 5 min in 4% paraformaldehyde in PBS, and covered with PBS/glycerin (9:1) containing DAPI. Ubiquitin (Ub), K63‐linked polyubiquitin (UbK63) and Cnot4 were detected essentially as described previously (Takáts et al., 2013). In brief, salivary glands were dissected in ice‐cold PBS then fixed with 4% formaldehyde in PBTX (0.1% Triton X‐100 in PBS for overnight at 4 °C). Samples were extensively washed with PBTX (3 × 15 min at RT) and then incubated in blocking solution (5% FCS in PBTX for 30 min at RT). Samples were then incubated with 1. monoclonal mouse anti‐Ub (clone A‐5: sc366553; Enzo) diluted 1:100, 2. monoclonal rabbit αUbK63 (clone JM09‐67; Invitrogen) diluted 1:100, 3. polyclonal rabbit αCnot4 (PA5‐101501; Invitrogen) diluted 1:100 in the blocking solution overnight at 4 °C. Salivary glands were then washed (3 × 15 min in PBTX at RT) and incubated in blocking solution again for 30 min at RT, followed by incubation with DyLight 488–conjugated goat α–rabbit (SA5‐10018; Thermo Fisher Scientific) diluted 1:600 in blocking solution for 3 h at RT. Washing steps were repeated, and samples were mounted with PBS/glycerol (9:1) containing DAPI. Images were taken at RT using a Carl Zeiss AxioImager M2 epifluorescent microscope equipped with an Apotome grid confocal unit and a led lamp, using AxioCam MRm camera Plan‐Apochromat 63 × NA = 1.4, EC Plan‐Neofluar 40 × NA = 0.75 objective and processed in Zeiss AxioVision SE64 Rel. 4.9.1 and Adobe Photoshop CS3 Extended.

### Transmission electron microscopy (TEM)

Progressive lowering temperature embedding and subsequent immunolabeling were performed as previously described (Lőrincz et al., 2014). In brief, salivary glands from control, *UAS‐Cnot4 I. RNAi and UAS‐Cnot4 II. RNAi* expressing, and *UAS‐Cnot4* overexpressing animals were dissected in PBS and fixed with 4% formaldehyde, 0.05% glutaraldehyde, and 0.2% tannic acid in phosphate‐buffered saline (PBS; 0.1 m, pH 7.4) overnight at 4 °C. Samples were then washed extensively with PBS, and free aldehyde groups were quenched with 50 mm glycine and 50 mm NH_4_Cl in PBS. Salivary glands were then postfixed in 1% uranyl acetate in 0.05 m maleate buffer (3 h at RT). Samples were then dehydrated in a graded series of ethanol as follows: 25% EtOH (10 min, 0 °C), 50% EtOH (10 min, 0 °C), 70% EtOH (10 min, −20 °C), 96% EtOH (20 min, −20 °C), and absolute EtOH (2 × 60 min, −20 °C). Next, salivary glands were infiltrated with pure LR White (Sigma‐Aldrich) containing 2% benzoyl peroxide as catalyst (24 h, −20 °C). Curing was performed using a homemade UV chamber (equipped with two 2 × 6‐W UV lamps) for 48 h at −20 °C. Ultrathin sections (80–90 nm) were cut and collected on nickel grids. Sections were viewed in a transmission electron microscope (JEM‐1011; JEOL) equipped with a digital camera (Morada; Olympus) using iTEM software 5.1 (Olympus).

### Statistical analysis

Fluorescence structures from original, unmodified single focal planes were quantified manually. Three to six cells were randomly selected for counting from pictures of control and RNAi salivary glands from 10 animals. In GFP‐ubiquitin and GFP‐Lamp1 experiments, glue granules with full or partial circles of GFP signal at the granule periphery were designated by us as positive for GFP‐Ubiquitin or GFP‐Lamp1. In glue‐GFP and glue‐DsRed experiments, all granules were counted and the number of glue‐GFP granules was proportioned to the number of all granules. We used GraphPad Prism 8 for data analysis. Tests indicated in the statistical table (Table S2).

## Results

### Ubiquitin is recruited to the surface of glue granules at the onset of developmentally programmed crinophagy

**Fig. 1:**
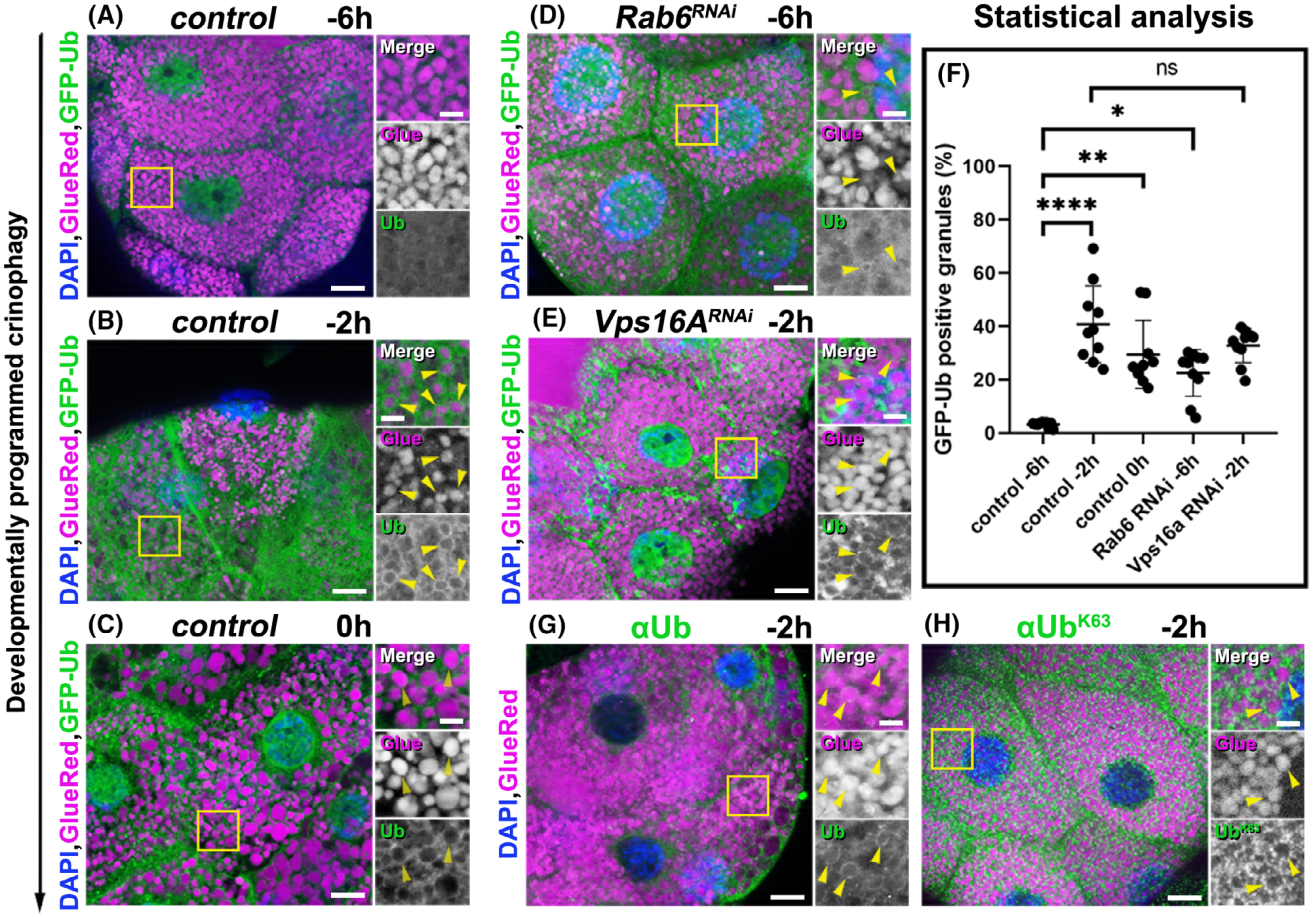
Glue granules are ubiquitinated during crinophagy. (A–C) Time‐course of ubiquitin localization during the naturally activated (developmentally programmed) crinophagy in the *Drosophila* larval and prepupal salivary gland cells co‐expressing glue‐DsRed and GFP‐tagged ubiquitin (GFP‐Ub). (A) Salivary gland cells from wandering larvae (−6 h RPF) contain many DsRed positive glue granules, and GFP‐Ub is seen in the cytoplasm and in the nucleus but not in the nucleolus. (B) In the late larval stage, 2 h before puparium formation (−2 h RPF), GFP‐Ub localizes on the membrane of multiple glue granules (yellow arrowheads) in the salivary gland cells. (C) A faint signal of GFP‐Ub is still visible on the membrane of glue granules crinosomes (yellow arrowheads) in the salivary glands from white prepupae (0 h RPF). (D–E) Silencing of selected genes in the *Drosophila* larval and prepupal salivary gland cells expressing glue‐DsRed and GFP‐Ub. (D) Knockdown of *Rab6* triggers premature recruitment of GFP‐Ub onto the membrane of a subset of immature glue granules (yellow arrowheads) in the salivary gland cells from the wandering animals (−6 h RPF), unlike in control cells (A). (E) Silencing of *Vps16A* does not impair the ubiquitin positivity of the membrane of glue granules (yellow arrowheads) in late larval salivary gland cells (−2 h RPF). (F) Quantification of data from panels A–C and D–E, *n* = 10 animals. Data are presented as mean ± SD. Statistical analysis was performed using Kruskal–Wallis and Dunn's multiple comparisons test. A *p* value of more than 0.05 was considered to be nonsignificant (^ns^ *p* > 0.9999) and a *p* value of less than 0.05 was significant (*P = 0.0333; **P = 0.0051; ****P < 0.0001). (G–H) Immunostaining of glue‐DsRed expressing salivary gland cells from the late larval developmental stage (−2 h RPF). (G) Immunohistochemical analysis clearly detects endogenous ubiquitin on the surface of secretory granules (yellow arrowheads). (H) An antibody specific for K63‐linked polyubiquitin (αUb^K63^) also labels the membrane of glue granules (yellow arrowheads). The boxed regions in panels (A–E and G–H) are shown as enlarged insets on the right side of each panel. Magenta and green channels of merged images are also shown separately as indicated. The boxed regions in panels (A–E and G–H) are shown enlarged. Scale bars of A–E and G–H panels 20 μm, insets 5 μm.

Based on previous studies on the role of ubiquitin in the selective macroautophagic degradation of various cellular organelles, such as mitochondria, chloroplasts, peroxisomes, small secretory vesicles [20, 24, 27], and vid vesicle‐vacuole fusion in yeast [30], we hypothesized that the crinophagic breakdown of the large, glue‐containing secretory granules in the late larval salivary gland cells of *Drosophila* may also be a ubiquitin‐dependent process. To test the localization of ubiquitin on secretory granules, we analyzed salivary glands from transgenic *Drosophila* that simultaneously expressed glue‐DsRed to label glue‐containing secretory granules and GFP‐tagged ubiquitin (GFP‐Ub), which we refer to as the glue‐ubiquitin reporter assay [32, 34]. This system allowed us to examine the localization of these reporter proteins during the developmentally activated crinophagy in the late larval and prepupal salivary gland cells. During the wandering larval phase (−6 h RPF—relative to puparium formation), the salivary gland cells exhibited numerous glue‐DsRed positive granules, and GFP‐ubiquitin was uniformly detected throughout the cytoplasm and the nucleus, excluding the nucleolus (Fig. 1A). During the late larval stage, when developmentally programmed crinophagy is typically activated in salivary gland cells [11], there was a pronounced and evident recruitment of GFP‐ubiquitin onto the surface of numerous granules containing glue‐DsRed (yellow arrowheads, Fig. 1B). The salivary glands of prepupal animals (0 h RPF) seemed to exhibit weaker GFP‐ubiquitin signal on the membrane of glue granules/crinosomes (pale yellow arrowheads on Fig. 1C). Importantly, panel F contains and shows the percentages of GFP‐Ub positive glue granules depicted in panels A–C. These findings reveal a robust association between the developmental stage characterized by elevated ubiquitin levels on the glue granules and the commencement of the naturally triggered, intense fusion process between the glue granules and lysosomes at −2 h RPF [11]. These findings raise the possibility that ubiquitination re‐routes secretory granules toward degradation, possibly acting as a signal to initiate crinophagy. Furthermore, our results suggest that ubiquitin only transiently associates with glue granule/crinosomal membranes, implying a sophisticated regulatory mechanism connected to the potential fates of glue granules in the late larval salivary gland cells.

### Disruption of endosome‐to‐TGN retrograde transport also causes the early ubiquitination of immature glue‐containing small secretory vesicles during developmental program‐independent crinophagy

In our recent paper, we employed genetic manipulation to trigger premature activation of crinophagy independent of the typical developmental process. We reported that disrupting the retrograde transport from endosomes to the trans‐Golgi network (TGN) leads to premature acidification and increased crinophagic degradation of the accumulated immature and small glue‐containing secretory vesicles within the salivary gland cells [3]. *Rab6* encodes a small GTPase that plays a critical role in the endosome‐to‐TGN retrograde transport pathway [39]. *Rab6* silencing in larval salivary gland cells from wandering animals (−6 h RPF) resulted in GFP‐ubiquitin recruitment onto the surface of small, immature glue‐DsRed positive vesicles (Fig. 1D,F). Importantly, ubiquitin did not normally localize to the membrane of glue granules at this developmental stage (Fig. 1A), implicating a potential role for ubiquitin in initiating developmental program‐independent crinophagy, similar to the developmentally regulated secretory granule–lysosome fusion.

### Ubiquitin is recruited to the membrane of glue granules independently of the endo‐lysosomal system

During normal cellular processes, ubiquitin plays diverse roles in various mechanisms, including endocytosis, different types of autophagy, and the maintenance of lysosomal membrane protein homeostasis [26, 28]. Ubiquitin primarily contributes to the functioning of the endo‐lysosomal system, thereby maintaining cellular homeostasis. Vps16A is an essential component of this system, as it is a shared subunit of the CORVET (class C cORe Vacuole/Endosome Tethering—miniCORVET in *Drosophila*) and HOPS (HOmotypic fusion and Protein Sorting) tethering complexes. The CORVET complex mediates the homotypic fusion of early endosomes, while HOPS is required for homo‐ and heterotypical lysosomal fusion events [40]. We showed previously that Vps16A is crucial for the fusion of secretory granules with lysosomes [11]. Since ubiquitin was shown to be recruited to the surface of endosomes and lysosomes [26, 29], we sought to establish the timing and mechanism of ubiquitin recruitment to the surface of glue granules during the induction of crinophagy. Therefore, we silenced *Vps16A* in the salivary gland cells of our glue‐DsRed and GFP‐ubiquitin expressing animals. Interestingly, we found that ubiquitin was still present on the membrane of glue granules when endosomal and lysosomal fusions were inhibited by loss of *Vps16A* in the gland cells from late larvae (−2 h RPF, Fig. 1E,F). This observation suggests that during the induction of crinophagy, ubiquitin is recruited directly to the surface of glue granules from the cytoplasm without the contribution of the endo‐lysosomal system.

### Endogenous ubiquitin is also present on the membranes of glue granules and forms K63‐polyubiquitin chains

In all previous experiments, we expressed GFP‐conjugated ubiquitin specifically in *Drosophila* salivary gland cells. We next used immunostaining to determine the localization of endogenous ubiquitin in the salivary gland cells expressing glue‐DsRed during the late larval stage. Consistent with our earlier findings, endogenous ubiquitin was recruited to the membrane of glue granules in the gland cells 2 h prior to puparium formation (−2 h RPF, Fig. 1G).

The appearance of diverse ubiquitin forms on molecules and cellular organelles raises the possibility that mono‐, multi‐, and/or polyubiquitin chains (with various linkages, such as K48, K63, and K11 polyubiquitin chains) may be present on glue granules [26]. The maturation of secretory granules relies heavily on the endosomal system, and endocytic trafficking and autophagic pathways are often regulated by Lysine‐63 (K63)‐linked polyubiquitin patterns on the surfaces of several cell organelles, such as mitochondria, endosomes, and lysosomes [26]. Based on these observations, we hypothesized that the possible type of ubiquitin found on glue granules may be K63‐linked polyubiquitin. Indeed, our immunostaining experiment with a chain‐specific antibody revealed that K63‐linked polyubiquitin was present on glue granule membrane (Fig. 1H).

In conclusion, glue granules acquire K63‐linked polyubiquitin chains, which likely serve as signals governing the direct fusion of these vesicles with late endosomes and lysosomes.

### The ubiquitin ligase Cnot4 is required for secretory granule–lysosome fusion and crinophagic degradation

During the late larval–prepupal developmental stage, the salivary glands undergo multiple significant morphological transformations associated with the completion of glue secretion. These changes encompass the developmentally regulated reconstruction of gland cell architecture during exocytosis, as well as the rapid removal and degradation of the remaining glue granules through extensive fusion with late endosomes and lysosomes. The initial molecular regulators identified in this process include the small GTPases Rab2, Rab7, and Arl8, the HOPS tethering complex, and the Syx13‐Snap29‐Vamp7/Ykt6‐containing SNARE complexes [11, 15, 16]. Furthermore, loss of the endosome‐to‐TGN recycling pathway components (such as *Rab6*, *Syx16*, and *Vps53*) leads to premature crinophagy in the salivary gland cells of *Drosophila* independently of the developmental program [3].

Ubiquitination, a dynamic post‐translational modification, is carried out by specialized enzymes. The activity of E3 enzymes facilitates the attachment and assembly of diverse ubiquitin patterns on the substrate molecule [24]. Motivated by the robust GFP‐ubiquitin labeling of glue granules in salivary gland cells during the late larval developmental stage, we conducted a targeted knockdown screen in transgenic *Drosophila* flies that co‐expressed glue‐GFP and glue‐DsRed to investigate crinophagy based on the quenching of pH‐sensitive GFP, but not pH‐resistant DsRed that stays fluorescent within the acidic lumen of lysosomes [11]. Positive controls in our screen included *dor*, *lt*, and *Vps11*, genes encoding RING (Really Interesting New Gene) domain‐containing subunits of the HOPS vesicle tethering complex, whose knockdown inhibited crinophagy as expected based on our previous papers [11].

**Fig. 2:**
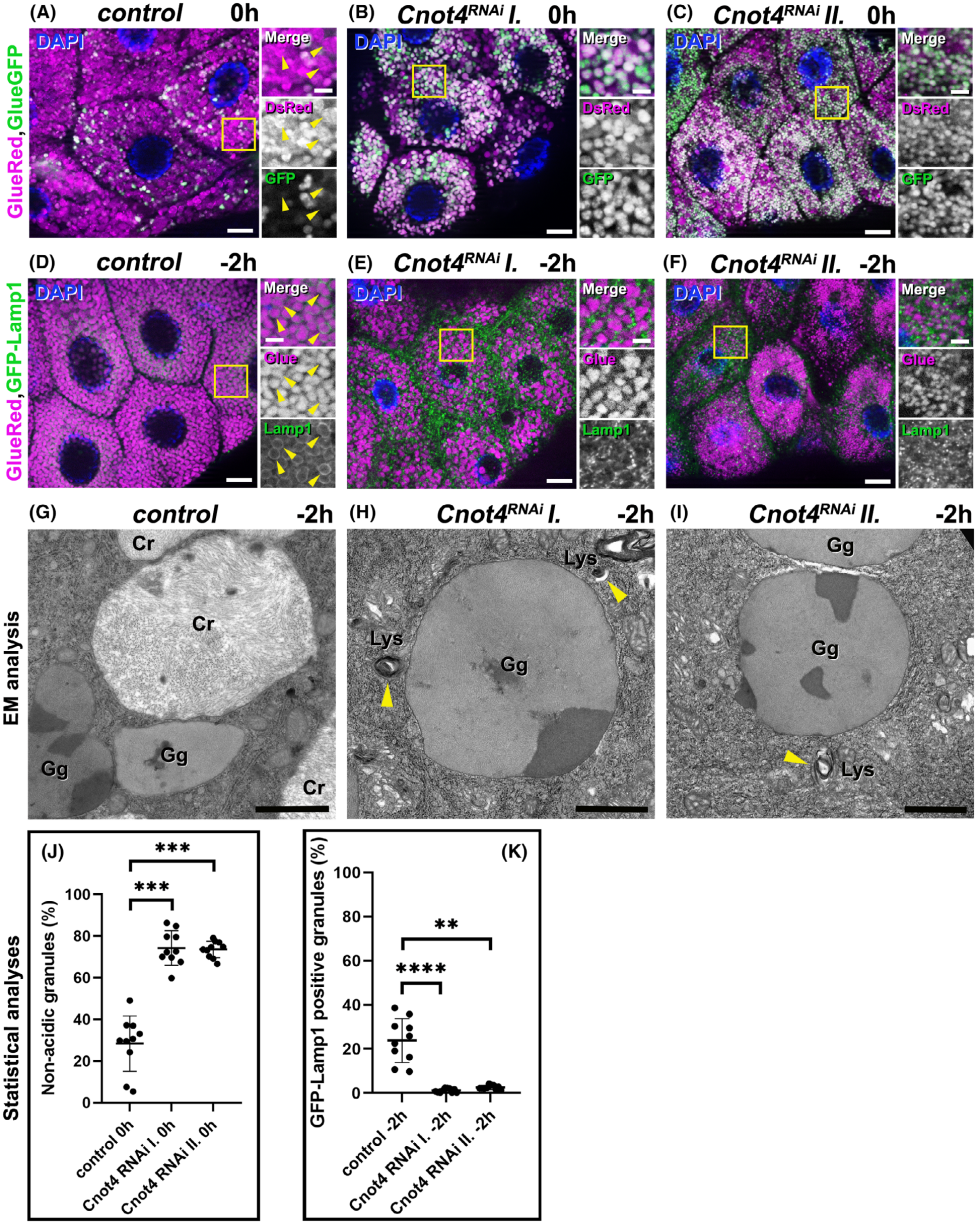
Loss of *Cnot4* impairs glue granule–lysosome fusion. (A–C) GFP is not quenched in glue‐containing secretory granules of prepupal (0 h RPF) salivary gland cells co‐expressing glue‐GFP/glue‐DsRed reporters if *Cnot4* is knocked down using independent RNAi constructs. Salivary gland‐specific independent knockdown of *Cnot4* (*RNAi I*—B) or (*RNAi II*—C) disrupts the developmentally programmed quenching of GFP fluorescence within glue granules compared with control cells (A, yellow arrowheads point to DsRed positive crinosomes). (D–F) Expression of *Cnot4 RNAi I* or *Cnot4 RNAi II* leads to impaired fusion of glue granules with late endosomes and lysosomes based on the glue‐DsRed and GFP‐Lamp1 reporters. The formation of GFP‐Lamp1 rings around glue‐DsRed positive secretory granules (yellow arrowheads) seen in control cells (D) is inhibited in salivary gland cells undergoing *Cnot4* (*Cnot4 RNAi I*—E) and (*Cnot4 RNAi II*—F) RNAi, indicating a fusion defect between glue granules and endo‐lysosomal vesicles. (G–I) Ultrastructural analysis confirms secretory granule‐late endosome/lysosome fusion defects in salivary gland cells from the late larval stage upon using different *Cnot4* knockdown lines: intact glue granules persist in *Cnot4 RNAi I* (H) or *Cnot4 RNAi II* (I) expressing cells. Both intact glue granules and crinosomes containing loose filamentous contents (Cr) are evident in wild‐type gland cells at ‐2 h RPF (G). The yellow arrowheads in panels (H) and (I) indicate lysosomes (Lys) near intact glue granules (Gg). Quantification of data from panels A–C (J) and D–F (K) were performed on samples from 10 animals. Data are presented as mean ± SD. Statistical analysis was performed using Kruskal–Wallis and Dunn's multiple comparisons test. A *p* value of less than 0.05 was significant (**P = 0.0064; ***P = 0.0003; ****P < 0.0001). Abbreviations: E3: ubiquitin ligase, Ub: Ubiquitin, K63 Ub: K63‐linked polyubiquitin, Sg: Secretory granule, Cr: Crinosome. The boxed regions in panels (A–F) are shown enlarged on the right side of each panel. Magenta and green channels of merged images are shown separately as indicated. Bars: (A–F) 20 μm, (G–I) 1 μm, insets 5 μm.

**Fig. 3:**
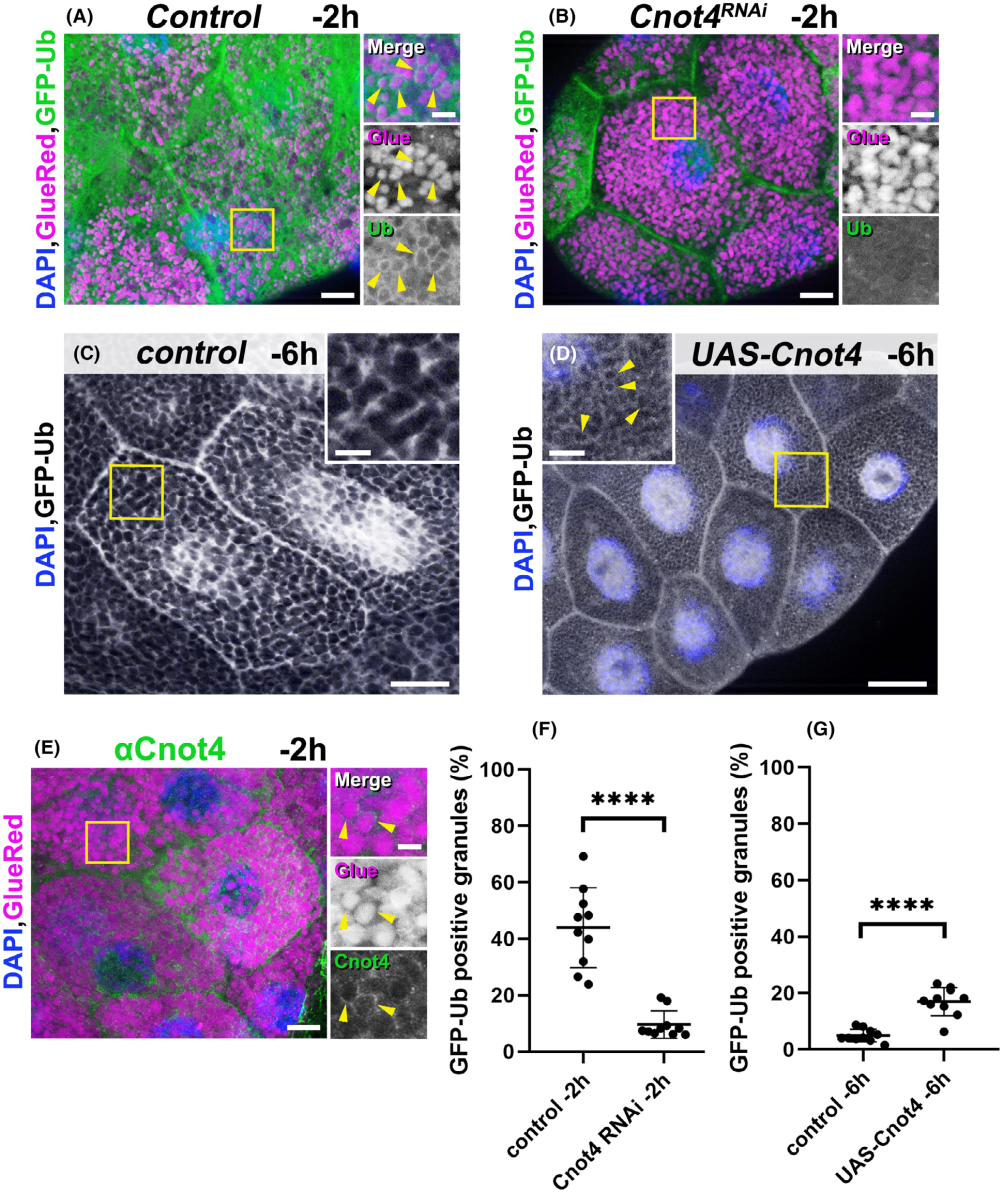
Cnot4 localizes to glue granule membranes and controls their ubiquitination. (A–F) Silencing of *cnot4* gene and overexpression of Cnot4 protein in the *Drosophila* larval and prepupal salivary gland cells expressing glue‐DsRed and GFP‐Ub. (B) Knockdown of *Cnot4* prevents ubiquitination of glue granules in late larvae (−2 h RPF), which is obvious in the gland cells of similarly staged control animals (A). (C and D) Overexpression of Cnot4 prevents GFP‐Ub localization on the surface of glue granules. Salivary gland cells from wandering animals (−6 h RPF) co‐expressing GFP‐Ub and Cnot4 already contain GFP‐ubiquitin‐positive glue granules (D, yellow arrowheads), compared with the control cells (C). (E) Immunostaining of glue‐DsRed expressing salivary gland cells from the late larval developmental stage (−2 h RPF). The ubiquitin ligase Cnot4 is present on the membrane of glue granules (yellow arrowheads) in the salivary gland cells from the late larval period (−2 h RPF). The boxed regions in panels (A–E) are shown as enlarged insets on the right side of each panel. Magenta and green channels of merged images are also shown separately as indicated. Scale bars of A‐E panels equal 20 μm, insets 5 μm. (F) Quantification of data from panels A–B, *n* = 10 animals. Data are presented as mean ± SD. (G) Quantification of data from panels C–D was performed on samples from 10 animals. Data are presented as mean ± SD. Statistical analyses were performed using Mann–Whitney test (F) and Unpaired *t‐*test with Welch's correction (G). A *p* value of less than 0.05 was significant (****P < 0.0001).

Our targeted screen for ubiquitin ligases (Table S1) relied on a previously established acidification assay, in which prepupal (0 h RPF) animals co‐express glue‐GFP and glue‐DsRed fusion proteins that label secretory cargo‐containing granules [11, 31, 34]. Crinosomes form in the control cells due to the fusion of glue‐GFP and glue‐DsRed‐containing secretory granules with acidic lysosomal compartments, and GFP fluorescence is rapidly quenched in the acidic crinosomal environment. Importantly, the glue‐DsRed component is less sensitive to low pH, allowing the degrading cargo to retain the DsRed signal. These markers enabled us to distinguish the intact secretory granules (GFP+, DsRed+) from acidic crinosomes (DsRed+ only)—Fig. 2A—[3, 11, 15]. From the several potential new hits (Table S1), we decided to study the crinophagic role of the gene *Cnot4*, which also encodes a RING type E3 enzyme. Remarkably, glue granules undergo substantial degradation during the late larval‐to‐prepupal transition, exactly when Cnot4 expression shows a 3‐fold upregulation in salivary gland cells during pupariation, underscoring the gene's potential involvement in glue granule crinophagy [11, 36, 37, 38]. Another reason for focusing on *Cnot4* was because its knockdown produced the most robust phenotype without perturbing the biogenesis (i.e., the size and number) of glue granules (RNAi 1), and no gland atrophy was observed either. Two independent *Cnot4* RNAi lines (*Cnot4* RNAi I and II, targeting different regions of this gene) exhibited a strong inhibition of GFP quenching (Fig. 2B,C and its statistical analysis on panel J), in contrast to the control cells that contained many crinosomes that are only positive for DsRed (Fig. 2A,J) at the prepupal developmental stage. Importantly, the knockdown of other subunits of the CCR4‐CNOT complex, such as *Not1* and *Rcd‐1*, did not perturb the intense acidification of glue granules (Fig. S1A–D).

The degradation of glue granules in the late larval/prepupal salivary gland cells occurs through the direct fusion of residual secretory granules and late endosomes–lysosomes. Given the putative regulatory function of *Cnot4* in lysosomal degradation [41], we examined whether knockdown of this gene also impairs secretory granule–lysosome fusion. To investigate the specific functions of *Cnot4* in crinophagy, we monitored the appearance of the lysosomal membrane marker GFP‐Lamp1 (Lysosome Associated Membrane Protein‐1) in the membrane of the DsRed‐containing granule granules [11, 15, 16, 33]. Control salivary gland cells from the late larval (−2 h RPF) developmental stage were full of glue granules surrounded by GFP‐Lamp1 (Fig. 2D and the corresponding statistical analysis presented in panel K), meaning that this lysosomal membrane protein was acquired into the membrane of glue granules via vesicle fusions. In contrast, small GFP‐Lamp1 positive structures accumulated in between the DsRed‐containing glue granules instead of forming rings encircling the granules in the salivary gland cells upon expression of *Cnot4* RNAi I or II, indicating impaired fusion with late endosomes and lysosomes (Fig. 2E, F and K). Furthermore, ultrastructural analysis of *Cnot4* silenced (*Cnot4* RNAi I or II) salivary gland cells identified unfused late endosomes and lysosomes near the glue granules, and glue granule content retained an immature (in comparison with crinosomes), not digested morphology compared with the control (Fig. 2G–I).

Our experiments on glue granule acidification and granule‐to‐lysosome fusion thus revealed the important role of Cnot4 in crinophagy in *Drosophila* salivary gland cells during the late larval and prepupal development.

### Cnot4 is required for the normal dynamics of glue granule ubiquitination during the late larval–prepupal transition of *Drosophila*

Cnot4 (CCR4‐NOT transcription complex, subunit 4) is an E3 enzyme that is a subunit of the CCR4‐NOT (Carbon Catabolite Repression 4—Negative On TATA‐less) deadenylase complex involved in mRNA degradation [42]. Interestingly, in *Drosophila* Cnot4 is not stably incorporated into the CCR4‐NOT complex, suggesting that it may possess additional independent functions [43].

We further investigated the role of *Cnot4* using our newly developed glue‐ubiquitin reporter system. Silencing the *Drosophila Cnot4* gene completely prevented the association of GFP‐ubiquitin with glue granules in late larval salivary gland cells (Fig. 3B and the corresponding statistical analysis presented in panel F), unlike what we saw in control cells at a similar developmental stage (Fig. 3A,F). Furthermore, overexpression of the Cnot4 protein in the salivary gland cells of wandering larvae led to premature ubiquitination of glue granules (Fig. 3D and its statistical analysis on panel G), in comparison with control cells (Fig. 3C,G). This observation indicates that Cnot4 plays a crucial role in the ubiquitination of glue granule membranes during developmentally programmed crinophagy in salivary gland cells.

Given the indispensable role of Cnot4 in the normal ubiquitination dynamics of glue granule membrane proteins and/or lipids during the late larval–prepupal transition, ubiquitin is likely a part of a precise regulatory system that directs glue granules toward crinophagic degradation.

### Cnot4 is recruited to the surface of glue granules during the late larval–prepupal transition

Cnot4 is a component of the CCR4‐NOT complex, which plays a crucial role in mRNA turnover. Although *Drosophila* Cnot4 (dCnot4) is not stably integrated into the CCR4‐NOT complex, this protein may modulate the ubiquitination of glue granule membranes either directly or through mRNA metabolism. To test this, we investigated the intracellular localization of endogenous Cnot4 using a Cnot4 antibody in *Drosophila* salivary glands. Importantly, endogenous Cnot4 was clearly observed on the surface of glue granules when developmentally programmed crinophagy was activated (−2 h RPF, Fig. 3E), suggesting that Cnot4 acts as a ubiquitin ligase that directly ubiquitinates the membrane of glue granules prior to their crinophagic degradation. Importantly, Cnot4 is localized to the glue granule membrane when intense crinophagic degradation begins in these cells.

Collectively, our loss of function and localization data support that Cnot4 plays a direct role in the formation of the ubiquitin pattern on the glue granule membrane, which could reroute glue granules toward crinophagic degradation.

### Overexpression of Cnot4 leads to early acidification and degradation of glue granules

**Fig. 4:**
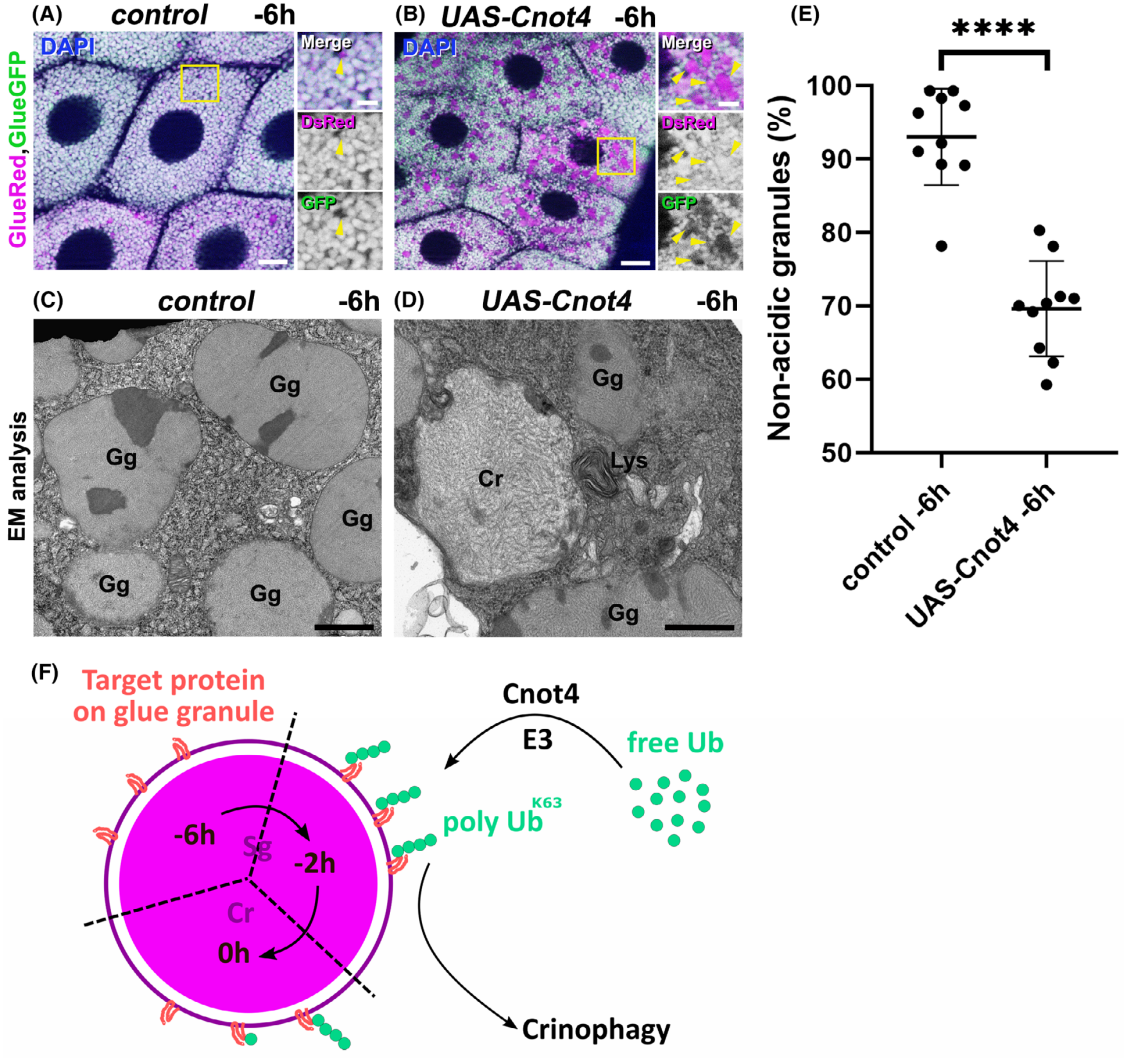
Overexpression of Cnot4 induces premature crinophagy in salivary gland cells. (A–B) Upon overexpression of Cnot4, acidification of glue granules occurs well before the developmental program in salivary gland cells from wandering animals based on the glue‐GFP and glue‐DsRed reporters. Salivary‐gland‐specific overexpression of Cnot4 causes early and intense quenching of GFP fluorescence within crinosomes (B, yellow arrowheads), compared with the control cells (A). (C–D) Ultrastructural analysis of salivary gland cells overexpressing Cnot4 (D) and controls (C). Intense crinosome formation is observed in Cnot4 overexpressing salivary gland cells from wandering animals (D), unlike in controls (C). Both intact glue granules and crinosomes containing loose filamentous contents (Cr) are evident in Cnot4 overexpressing gland cells at ‐2 h RPF (D), whereas control cells only contain intact glue granules at this stage (C). (E) Quantification of data from panels A and B was performed on samples from 10 animals. Data are presented as mean ± SD. Statistical analysis was performed using Mann–Whitney test Statistical analysis was performed using Mann–Whitney test. A *p* value of less than 0.05 was significant (****P < 0.0001). (F) The model depicts that glue granule designation for crinophagic degradation involves ubiquitin, and its conjugation to the surface of glue granules is mediated by the ubiquitin ligase Cnot4. Abbreviations: Gg: Glue granule, Cr: Crinosome, Lys: lysosome. The boxed regions in panels (A and B) are shown enlarged on the right side of each panel. Magenta and green channels of merged images are shown separately as indicated. Bars: (A and B) 20 μm, insets 5 μm, (C and D) 2 μm.

Ubiquitin ligases are involved in the regulation of different vesicular trafficking pathways, such as lysosomal/vacuolar membrane protein degradation via recycling of membrane proteins of these organelles. An earlier published study clearly identified the premature induction of vacuolar lysine transporter YPQ1 recycling and degradation if the regulator protein (an E3 enzyme Rsp5) of this pathway is overexpressed in the yeast cells [28]. Therefore, we wanted to know whether the overexpression of Cnot4 ubiquitin ligase also causes early acidification and degradation of glue‐containing secretory granules. Based on the premature GFP‐ubiquitin positivity of glue granules induced by Cnot4 overexpression compared with the control cells (Fig. 3C,D and its statistical analysis on panel G), we also tested whether Cnot4 gain‐of‐function can induce premature crinophagy in the salivary glands of animals co‐expressing glue‐GFP and glue‐DsRed. Strikingly, compared with the control cells (Fig. 4A, C and E), overexpression of Cnot4 in salivary gland cells of wandering animals indeed led to premature acidification and degradation of a subset of glue granules (Fig. 4B, D and E). It is important to note that our ultrastructural investigations (Fig. 2G and Fig. 4D) also support that the acidic (only glue‐DsRed‐positive) structures seen in Fig. 2A and Fig. 4B correspond to crinosomes [11]. These crinophagy modulating effects of Cnot4 overexpression likely stem from modulation of glue granule ubiquitination (Fig. 3D and G).

### Ubiquitin is not required for glue release from the salivary gland, but premature ubiquitination and crinophagy induced by Cnot4 overexpression impairs secretion

The molecular composition of ubiquitin modifications on organelles can induce diverse cellular processes, such as the initiation of autophagic degradation or the regulation of vesicular transport pathways [26]. The possible fates of glue‐containing secretory granules after their maturation may be exocytosis or crinophagic degradation through intense late endosomal‐lysosomal fusions. We thus examined whether Cnot4 also plays a role in secretion of glue from the salivary gland. In our previous paper, we established that crinophagy is not necessary for the secretory function of *Drosophila* salivary gland cells [11]. Using the glue‐DsRed transgene, we examined the secretory activity of salivary glands in different genetic backgrounds. Consistent with our previous results, neither *Vps16a* nor our independent *Cnot4* RNAi knockdowns disrupted glue secretion (Fig. S2A–D, and the corresponding statistical analysis presented in panel G). It is important to note that the overexpression of Cnot4 protein using UAS‐Cnot4 did perturb the secretory function of the treated salivary gland cells (Fig. S2E,G). It is similar to what we found in our previous study, in which we identified the role of endosome‐to‐TGN retrograde transport genes *(Syntaxin 16—Syx16, Vps53)* in crinophagy, where silencing of *Syx16* or *Vps53* also caused early glue granule acidification, accumulation of small glue granules, and secretory defects in the gland cells [3]. Our results thus clearly show that Cnot4 has a similar role in the modulation and regulation of secretory granule crinophagic degradation.

We wanted to investigate the role of ubiquitin on the membrane of glue granules, so we next tested whether ubiquitin takes part in the designation of glue granules to the exocytotic pathway. To this end, we examined secreting salivary glands that simultaneously expressed GFP‐ubiquitin and LifeAct‐Ruby, the marker of secretory granule fusion with the apical plasma membrane [8, 9]. We observed numerous LifeAct‐Ruby‐positive granular structures localized near the apical membranes of the cells, which were associated with the expanding glandular lumen. Of note, GFP‐ubiquitin did not colocalize with LifeAct‐Ruby on the surface of the glue granules undergoing secretion, suggesting that ubiquitin does not play a prominent role in the exocytosis of these granules (Fig. S2F and the corresponding statistical analysis presented in panel H).

## Discussion

During the intracellular transport of cellular organelles and molecules, these components are precisely delivered to their required locations at the right time. Consequently, these processes necessitate intricate regulatory systems, among which ubiquitin and its regulatory enzymes: ubiquitin ligases and proteases represent key elements. Several vesicular transport pathways rely on ubiquitination, including endocytosis, cargo sorting, intraluminal vesicle formation during multivesicular body (MVB) biogenesis, modulation of cytoskeletal components, and autophagic mechanisms [29].

Crinophagy, a noncanonical autophagic process, involves the fusion of secretory granules with vesicles of the endo‐lysosomal system. The well‐established function of this process is the degradation and recycling of obsolete secretory contents [11, 12], and recent investigations implicated additional roles for crinophagy and crinophagy‐like mechanisms, including antigen generation in pancreatic β cells [44, 45], secretory granule quality control in exocrine and endocrine cells [3, 7], and a possible contribution to the secretory granule maturation [4, 6].

This study focused on the signal triggering crinophagy, which targets the unreleased glue granules in the late larval salivary gland cells of *Drosophila* after the burst of secretion. We found that the unreleased glue granules become ubiquitin‐positive, and this molecular pattern contains K63‐linked polyubiquitin, the most common ubiquitination signal in vesicular trafficking pathways [26, 29]. Furthermore, we also identified the E3 enzyme Cnot4, which facilitates the demolition of glue granules during development. In line with this, we showed that endogenous Cnot4 localizes to the surface of glue granules, and it is required for their ubiquitination (Fig. 4F). Moreover, overexpression of Cnot4 induces premature glue granule ubiquitination and crinophagy, which impairs proper secretion. These point to the critical role of Cnot4 in the regulation of crinophagy.

Future investigations will delineate the precise mechanism by which ubiquitination of glue granules facilitates fusion between secretory granules and lysosomes. A key question concerns the identity of the molecule on the glue granule membrane that serves as the ubiquitination target for Cnot4. It is noteworthy that ubiquitin conjugation extends beyond proteins to encompass carbohydrates, lipids, and nucleic acids [25]. Another critical issue is the mechanism whereby the ubiquitin signal initiates crinophagy. Previous studies have demonstrated that endosomal machinery and retrograde transport from endosomes to the TGN are essential for glue granule maturation in the *Drosophila* larval salivary gland cells during postembryonic development [1, 3]. In particular, Hao et al. characterized an E3 enzyme complex composed of MAGE‐L2 and Trim27 that regulates endosomal protein trafficking and recycling via K63‐linked polyubiquitin chains on the Wash endosomal protein [46, 47]. Given the central role of endosomes in glue granule maturation, crinophagic degradation of these granules may proceed through a recycling pathway for secretory granule‐specific membrane proteins involving K63‐linked polyubiquitination. Such a pathway would require a ubiquitin ligase complex incorporating Cnot4 and specialized for crinophagy. Here, we first demonstrate that Cnot4 ubiquitin ligase localizes to the surface of glue granules in salivary gland cells at the onset of metamorphosis (Fig. 3E). A major unresolved question involves the recruitment mechanism that targets Cnot4 to the glue granule surface. Numerous examples show that ubiquitin ligases lacking transmembrane domains can associate with organelle membranes. For instance, in yeast, the ubiquitin ligase Rsp5—which directs vacuolar membrane protein sorting and degradation—is anchored to the vacuolar surface via the transmembrane adaptor Ssh4 [48, 49].

Taken together, our findings support that ubiquitin has a broad role in multiple secretory granule–lysosome fusion events, including both developmentally programmed and prematurely triggered (in *Rab6*‐silenced salivary gland cells) forms of crinophagy. Our research provides new molecular insights into crinophagy, advancing our understanding of this process under both normal and pathological circumstances.

## Author contributions

TC designed research with input from PL and GJ. TC, AD, AK, AJ, HL‐D, and PL performed experiments. TC evaluated data. TC and GJ acquired funding. TC, PL, and GJ wrote the paper with comments from all authors.

## Funding

This work was supported by the National Research, Development, and Innovation Office (NRDIO) (PD135447 to TC, PD145868 to AJ, and K146634 to GJ), the New National Excellence Program of the Ministry for Innovation and Technology from the source of the National Research, Development and Innovation Fund (ÚNKP‐23‐5‐ELTE‐603 to TC) and Momentum/Lendület Grant of Hungarian Academy of Sciences and János Bolyai Research Scholarship of the Hungarian Academy of Sciences (LP2023‐6 to GJ and BO/00023/21/8 to TC).

## Supporting information


**Fig. S1**. Silencing genes encoding subunits of the CCR4‐CNOT complex other than Not1 does not influence glue granule acidification.
**Fig. S2**. Ubiquitin is not required for normal glue release from the salivary gland, while premature crinophagy induced by Cnot4 overexpression prevents glue secretion.
**Table S1**. Results of the RNAi screen of the *Drosophila* E3 enzyme encoding genes.
**Table S2**. Summary of statistical analyses.

## Data Availability

All data supporting the conclusions of this article are included within the article and its supporting information files. No large‐scale datasets requiring public repository deposition were generated in this study.
