# Supplementary material for: Ubiquitination of secretory granules promotes their crinophagic degradation in Drosophila
Source: FEBS Lett. 2026 May 31;600(12):1729–43. doi: 10.1002/1873-3468.70376 (PMC13284801; doi:10.1002/1873-3468.70376)
Supplement: Supplementary file 1 — Fig. S1. Silencing genes encoding subunits of the CCR4‐CNOT complex other than Not1 does not influence glue granule acidification. Fig. S2. Ubiquitin is not required for normal glue release from the salivary gland, while premature crinophagy induced by Cnot4 overexpression prevents glue secretion. Table S1. Results of the RNAi screen of the Drosophila E3 enzyme encoding genes. Table S2. Summary of statistical analyses. [file FEB2-600-1729-s001.pdf]

Title

Ubiquitination of secretory granules promotes crinophagic degradation in *Drosophila*

Authors

Tamás Csizmadia[1✉], Anna Dósa[1,2], Asha Kiran Maddali[3,4], András Jipa[4], Hajnalka Laczkó-Dobos[4], Péter Lőw[1*] and Gábor Juhász[1,4*✉]

Affiliations

[1]Department of Anatomy, Cell and Developmental Biology, Eötvös Loránd University, Budapest, Hungary

[2]Research Institute of Molecular Pathology (IMP), Vienna BioCenter PhD Program

[3]Heidelberg University Biochemistry Center (BZH), Heidelberg, Germany

[4]Institute of Genetics, HUN-REN Biological Research Centre, Szeged, Hungary

[*]These authors contributed equally

Contact information:

[✉]Corresponding authors: tamas.csizmadia@ttk.elte.hu; gabor.juhasz@ttk.elte.hu

Running title:

Glue granule ubiquitination triggers crinophagy

SUPPLEMENTARY FIGURES AND LEGENDS

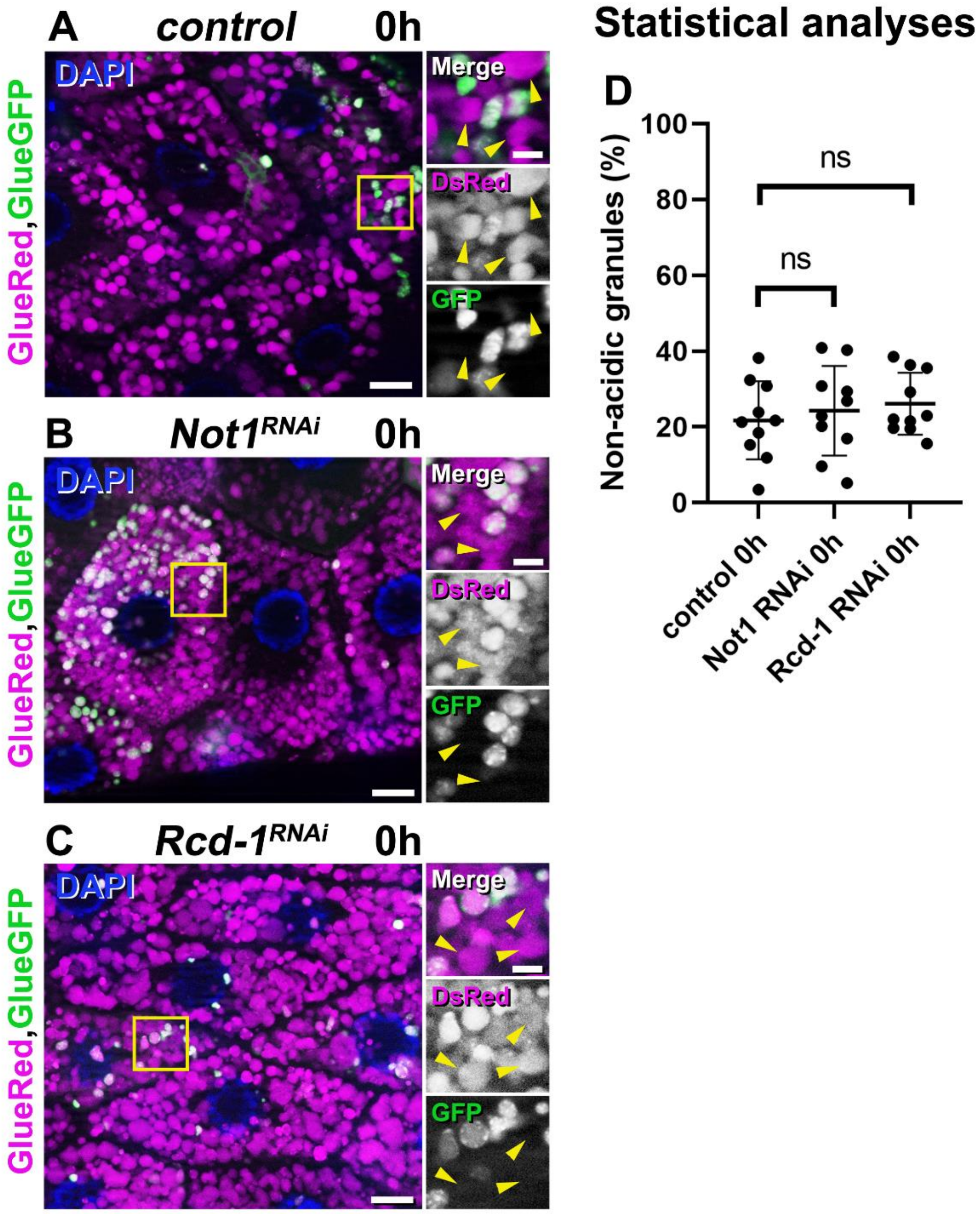

Figure S1. Silencing genes encoding subunits of the CCR4-CNOT complex other than Cnot4 does not influence glue granule acidification

(**A-D**) Prepupal salivary gland cells co-expressing glue-GFP and glue-DsRed reporters and transgenic RNA interference constructs for genes encoding subunits of the CCR4-CNOT complex. In control cells, the majority of glue granules transform exclusively into DsRed-positive crinosomes (**A**). Salivary gland-specific knockdown of *Not1* (**B**) or *Rcd-1* (**C**) did not

disrupt the developmentally programmed quenching of GFP fluorescence within glue granules: these are similar to control cells (**A,** yellow arrowheads). (**D)** Quantification of data from **A-C**, n=10 animals. Data are presented as mean ± SD. Statistical analysis was performed using Kruskal-Wallis and Dunn's multiple comparisons test. A p value of more than 0.05 was considered to be non-significant - ^{ns}p>0.9999 (*control–Not1*^{RNAi}) and ns=08038 (*control – Rcd-1*^{RNAi}). The boxed regions in panels (**A-C**) are shown as enlarged insets on the right side of each panel. Magenta and green channels of merged images are also shown separately as indicated. Scale bars of A-C panels equal 20 µm, insets 5 µm.

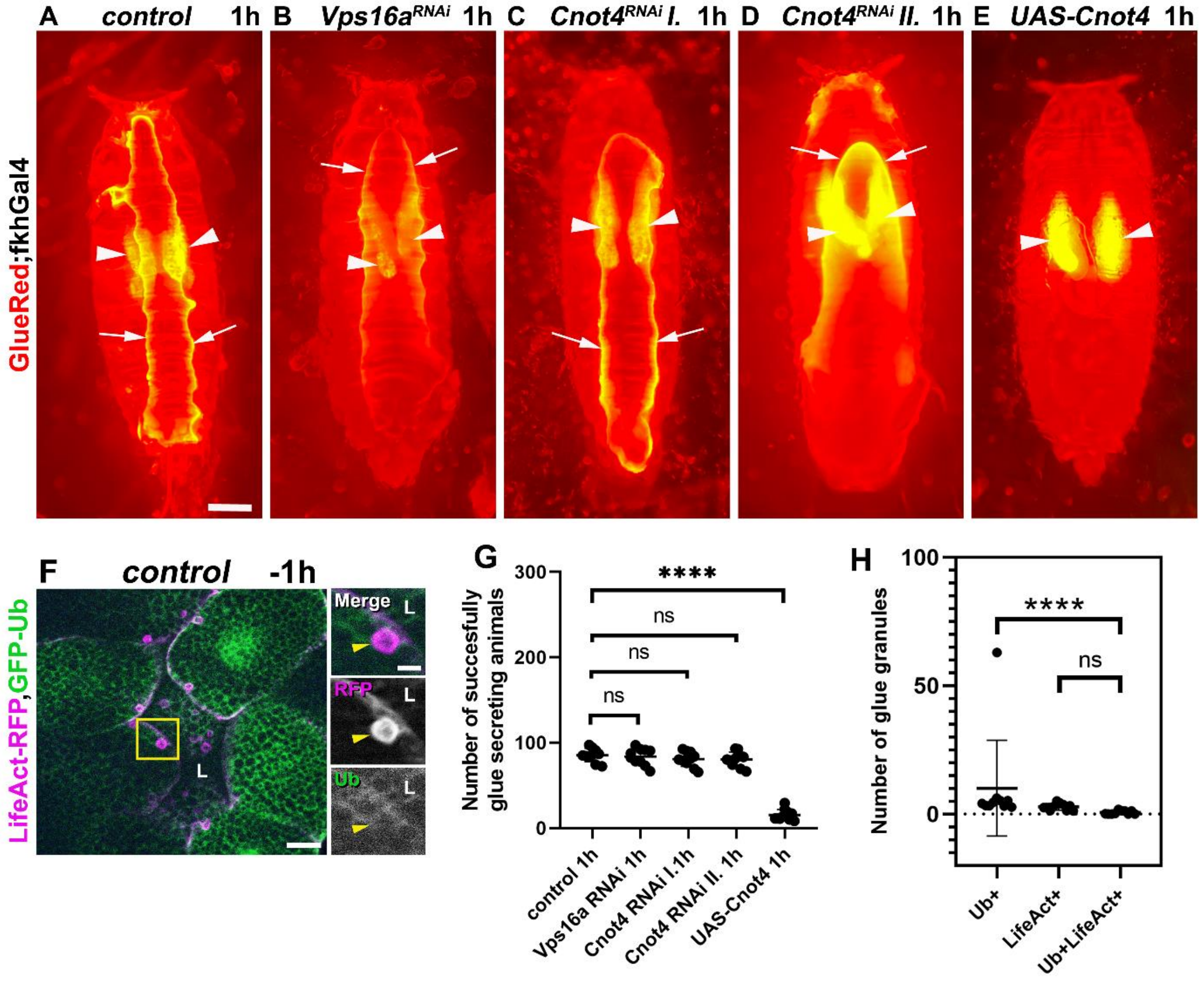

Figure S2. Ubiquitin is not required for normal glue release from the salivary gland, while premature crinophagy induced by Cnot4 overexpression prevents glue secretion

(**A-E**) Secretion assays using glue-DsRed animals in different genetic backgrounds. (**A**) Control animals display an obvious glue-DsRed patch under the prepupa (white arrows denote the border of the glue patch), which indicate the successful glue secretion and release from the salivary gland (glands are marked by white arrowheads). Silencing of the crinophagic gene *Vps16a* (**B**) or *Cnot4 (Cnot4 RNAi I* - **C** *and II* - **D***)* did not perturb the salivary gland's secretory process. Importantly, the overexpression of Cnot4 protein clearly results in strong defects in glue release from the gland (**E**). (**G**) Quantification of data from panels A-E (**G**) was performed on samples from 300 animals. Data are presented as mean ± SD. Statistical analysis was performed using Kruskal-Wallis and Dunn's multiple comparisons test. A p value of more than 0.05 was considered to be non-significant - ^{ns}p>0.9999 (*control – Vps16A*^{RNAi}*; control – Cnot4*^{RNAi1} *and control – Cnot4*^{RNAi2}). A p value of less than 0.05 was significant (****p<0.0001). (**F**) GFP-Ubiquitin (in green) does not colocalize with the exocytotic marker LifeAct-Ruby (in red) on glue granules in the salivary gland cells from the secretion period. The large LifeAct-Ruby positive structures are glue granules undergoing exocytosis [8,9], but they exhibit no GFP-Ub signal (yellow arrowheads). Sec: secretion period, L: lumen. (**H**) Quantification of data of panel **F**, n=6-8 animals. Data are presented as mean ± SD. Statistical analysis was performed using Kruskal-Wallis and Dunn's multiple comparisons test;. A p value of more than 0.05 was considered to be non-significant (^{ns}p=0.1820) and a p value of less than 0.05 was significant (****p<0.0001).

The boxed region in panel (**F**) is shown as enlarged insets on the right side of the panel. Magenta and green channels of merged images are also shown separately as indicated. Scale bars of A-E panels equal 0.25 mm, F panel 20 µm, insets 5 µm.

SUPPLEMENTARY TABLES

Table S1. Results of the RNAi screen of the *Drosophila* E3 enzyme encoding genes.

**Potential *Drosophila* E3 Ub ligases examined in this study**						
Type of E3 enzyme/domain	***Number***	***Name or CG number***	***RNAi line***	***Source***	***Crinophagy status***	***Other comment***
**Goliath type**	1	gol	2679R-2	NigFly	no effect	—
	2	gzl	10277R-3	NigFly	no effect	—
**HECT type**	3	CG3356	3356R-1	NigFly	no effect	—
	4	CG4238	v41982	VDRC	no effect	—
	5	CG5087	5087R-3	NigFly	no effect	—
	6	CG42797	3003Ra-4	NigFly	no effect	—
	7	ctrip	HMS00322	NigFly	no effect	—
	8	HERC2	11734R-3	NigFly	no effect	—
	9	Herc4	v37220	VDRC	no effect	—
	10	HUWE1	8184R-1	NigFly	no effect	—
	11	hyd	9484R-2	NigFly	no effect	small salivary glands and granules
	12	Nedd4	7555R-3	NigFly	no effect	—
	13	Smurf	4943R-2	NigFly	no effect	—
	14	Su(dx)	4244R-1	NigFly	no granules	very small salivary gland
	15	Ube3a	31972	BDSC	no effect	—
	16	Ufd4	5604R-3	NigFly	persisting GFP	—
**IAP type**	17	Diap1	12284R-2	NigFly	no effect	—
	18	Diap2	8293R-1	NigFly	no effect	—
**Kcmf1 type**	19	CG3526	v26214	VDRC	no effect	—
	20	CG15286	15286R-3	NigFly	no effect	—
	21	CG31642	HMJ21895	NigFly	no effect	—
	22	CG31835	31835R-2	NigFly	no effect	—
	23	CG42585	HMJ23964	NigFly	no effect	—
	24	Kcmf1	11984R-2	NigFly	no granules	—
**Other type**	25	CG3894	42618	BDSC	no effect	—
	26	CG7326	7326R-1	NigFly	no effect	—
	27	CG13994	13994R-2	NigFly	no effect	—
	28	CG14646	14646R-4	NigFly	persisting GFP	variable effect
	29	I-3	v45386	VDRC	no effect	—
	30	Neurl4	44094	BDSC	no effect	—
	31	poe	14472R-1	NigFly	no effect	—
	32	Bre1	HMJ22277	NigFly	no effect	—

Other Ring types	33	Cbl	7037R-1	NigFly	no effect	—
	34	CG1317	HMJ21301	NigFly	no effect	—
	35	CG1909	HMC02933	NigFly	no effect	—
	36	CG2617	2617R-1	NigFly	no effect	—
	37	CG2617	HMJ21915	NigFly	no effect	—
	38	CG2681	2681R-1	NigFly	no effect	—
	39	CG2926	2926R-2	NigFly	persisting GFP	very small gland
	40	CG2991	2991R-3	NigFly	no effect	—
	41	CG4080	4080R-2	NigFly	no effect	—
	42	CG4325	v34829	VDRC	no effect	—
	43	CG4813	4813R-1	NigFly	persisting GFP	small granules
	44	CG5071	5071R-1	NigFly	no effect	—
	45	CG5334	5334R-1	NigFly	no effect	—
	46	CG5347	5347R-1	NigFly	persisting GFP	small granules
	47	CG5382	v101394	VDRC	no effect	—
	48	CG5555	5555R-2	NigFly	persisting GFP	—
	49	CG6752	6752R-1	NigFly	no effect	—
	50	CG6923	6923Ra-3	NigFly	no effect	—
	51	CG7376	v35222	VDRC	no effect	—
	52	CG7694	7694R-1	NigFly	no effect	—
	53	CG8141	HMJ23674	NigFly	no effect	—
	54	CG8910	8910R-3	NigFly	persisting GFP	variable effect, small granules
	55	CG8974	not available	—	—	—
	56	CG9014	9014R-1	NigFly	no effect	—
	57	CG9855	HMJ23874	NigFly	no effect	—
	58	CG9941	v29596	VDRC	no effect	—
	59	CG10761	v5474	VDRC	no effect	—
	60	CG10916	HMJ23868	NigFly	no effect	—
	61	CG11360	11360R-4	NigFly	no effect	—
	62	CG11414	11414R-3	NigFly	no effect	—
	63	CG12099	v18734	VDRC	no effect	—
	64	CG12477	v31944	VDRC	no effect	—
	65	CG13025	13025R-2	NigFly	no effect	—
	66	CG13344	13344R-2	NigFly	no effect	—
	67	CG13442	HMS01518	NigFly	no effect	—
	68	CG13481	v103527	VDRC	no effect	—
	69	CG13605	v105112	VDRC	no effect	—
	70	CG14435	14435R-4	NigFly	no granules	very small salivary gland
	71	CG14983	HMJ23801	NigFly	no effect	—

	72	CG15011	15011R-3	NigFly	no effect	—
	73	CG15141	15141R-1	NigFly	no effect	—
	74	CG15814	v30430	VDRC	no effect	—
	75	CG16781	v7020	VDRC	no effect	—
	76	CG17019	17019R-6	NigFly	no effect	—
	77	CG17048	v8780	VDRC	no effect	—
	78	CG17260	17260R-1	NigFly	no effect	—
	79	CG17329	v19171	VDRC	no effect	—
	80	CG17717	17717R-2	NigFly	no effect	—
	81	CG17721	v6036	VDRC	no effect	—
	82	CG17991	17991R-1	NigFly	no effect	—
	83	CG31807	31807R-2	NigFly	no effect	—
	84	CG32369	64028	BDSC	no effect	—
	85	CG32581	not available	—	—	—
	86	CG32847	v48423	VDRC	no effect	—
	87	CG32850	HMJ22085	NigFly	no effect	—
	88	CG33552	HMJ23596	NigFly	no effect	—
	89	CG34289	HMJ21306	NigFly	no effect	—
	90	CG34308	not available	—	—	—
	91	CG34375	13835R-2	NigFly	no effect	—
	92	Cnot4	31716R-1	NigFly	persisting GFP	—
	93	Cnot4	v10850	VDRC	persisting GFP	—
	94	d4	2682R-1	NigFly	persisting GFP	—
	95	dgrn	10981R-3	NigFly	no effect	—
	96	dnr1	12489R-1	NigFly	persisting GFP	small granules
	97	dor	3093R-2	NigFly	persisting GFP	—
	98	dx	3929R-1	NigFly	no effect	—
	99	elfless	15150R-1	NigFly	no effect	—
	100	elgi	17033R-3	NigFly	no effect	—
	101	Fancl	12812R-1	NigFly	no effect	—
	102	Ltn1	9268R-3	NigFly	no effect	—
	103	Mat1	7614R-1	NigFly	no effect	—
	104	mdlc	4973R-1	NigFly	no effect	—
	105	Hakai	2LG-0654	NigFly	no effect	—
	106	hiw	28031	BDSC	no effect	—
	107	Iru	11982R-3	NigFly	no effect	—
	108	CG4195	4195R-1	NigFly	no effect	—
	109	ImgA	2LG-0313	NigFly	no effect	—
	110	Lpt	25994	BDSC	no effect	—

	111	lt	18028R-2	NigFly	persisting GFP	—
	112	Mi-2	HMS00301	NigFly	no effect	—
	113	mib1	27320	BDSC	no effect	—
	114	mib2	HMJ21843	NigFly	no effect	—
	115	Mkrn1	v34373	VDRC	no effect	—
	116	msl-2	31627	BDSC	no effect	—
	117	Mul1	1134R-2	NigFly	no effect	—
	118	Mura	GL00121	NigFly	no effect	—
	119	neur	11988R-1	NigFly	no effect	—
	120	nopo	v22013	VDRC	no effect	—
	121	Nse1	2LG-0755	NigFly	no effect	—
	122	Pex2	7081R-2	NigFly	no effect	—
	123	Pex10	v46613	VDRC	no effect	—
	124	Pex12	3639R-1	NigFly	no effect	—
	125	Pli	5212R-3	NigFly	no effect	—
	126	POSH	4909R-1	NigFly	no effect	—
	127	Psc	3886R-1	NigFly	no effect	—
	128	qin	HMJ21020	NigFly	no effect	—
	129	Rbpn-5	4030R-3	NigFly	no effect	—
	130	Rchy1	HMJ22014	NigFly	no effect	—
	131	Rnf146	8786R-4	NigFly	no effect	—
	132	roq	HMJ21890	NigFly	no effect	—
	133	Sce	5595R-1	NigFly	persisting GFP	very few and small granules
	134	sina	9949R-1	NigFly	no effect	—
	135	sinah	13030R-2	NigFly	no effect	—
	136	sip3	1937R-3	NigFly	no effect	—
	137	snama	3231R-3	NigFly	no effect	—
	138	snky	HMJ21388	NigFly	no effect	—
	139	stc	HMS02768	NigFly	no effect	—
	140	Su(z)2	HMS00281	NigFly	no effect	—
	141	Topors	15104R-2	NigFly	no effect	—
	142	Traf6	10961R-1	NigFly	no effect	—
	143	Trc8	v4449	VDRC	no effect	—
	144	trx	HMS00580	NigFly	no effect	—
	145	Ubr1	9086R-3	NigFly	no effect	—
	146	Ubr3	v22901	VDRC	no effect	—
	147	Unk	57026	BDSC	no effect	—
	148	Vps8	10144R-2	NigFly	no effect	—
	149	Vps11	v24731	VDRC	persisting GFP	—
	150	ari-1	29416	BDSC	no effect	—
**Ring btw Ring**	151	ari-2	5709R-1	NigFly	no effect	—
	152	CG12362	12362R-1	NigFly	no effect	—

	153	CG33144	64033	BDSC	no effect	—
	154	LUBEL	11321R-3	NigFly	no effect	—
	155	park	10523R-2	NigFly	no effect	—
**Roc types**	156	Roc1a	HMS00353	NigFly	no effect	—
	157	Roc1b	31067	BDSC	no effect	—
	158	Roc2	v28103	VDRC	no effect	—
**Trim types**	159	bon	27047	BDSC	no effect	—
	160	CG8419	v107626	VDRC	no effect	—
	161	mei-P26	12218R-1	NigFly	no effect	—
	162	tn	HMS02508	NigFly	no effect	—
	163	Trim9	31721R-2	NigFly	no effect	—
**U-box types**	164	CG2218	2218R-4	NigFly	no effect	—
	165	CG6197	6179R-1	NigFly	no effect	—
	166	CG7747	v44854	VDRC	no effect	—
	167	CG9934	HMJ21044	NigFly	no effect	—
	168	CG11070	v110416	VDRC	no effect	—
	169	Prp19	5519R-1	NigFly	no effect	—
	170	STUB1	5203R-3	NigFly	no effect	—

Table S2. Summary of statistical analyses.

Table of statistics							
**Figure**	**Panel**	**Dev.stage and Genotype**	**Mean**	**SD**	**P-value**	**Pairs**	**Test**
**1.**	**F**	*control -6h*	3,244	1,018			Kruskal-Wallis + Dunn's multiple comparisons test
		control -2h	40,78	14,41	0,0051	control -6h - control -2h	
		control 0h	29,45	12,75	<0,0001	control -6h - control 0h	
		Rab6 RNAi -6h	22,58	8,748	0,0333	control -6h - Rab6 RNAi -6h	
		Vps16A RNAi -2h	32,79	6,443	>0,9999	control -2h - Vps16A RNAi -2h	
**2.**	**J**	*control 0h*	28,34	13,28			Brown-Forsyte and Welch ANOVA + Dunnett's T3 mutiple comparisons test
		Cnot4 RNAi I. 0h	74,24	8,261	<0,0001	control 0h - Cnot4 RNAi I. 0h	
		Cnot4 RNAi II. 0h	73,56	2,612	<0,0001	control 0h - Cnot4 RNAi II. 0h	
	**K**	*control -2h*	23,86	9,956			Kruskal-Wallis + Dunn's multiple comparisons test
		Cnot4 RNAi I. -2h	1,175	0,8857	<0,0001	control -2h - Cnot4 RNAi I. -2h	
		Cnot4 RNAi II. -2h	2,595	0,9497	0,0064	control -2h - Cnot4 RNAi II. -2h	
**3.**	**F**	*control -2h*	43,97	14,15			Mann-Whitney test
		Cnot4 RNAi (I.) -2h	9,639	4,83	<0,0001	control -2h - Cnot4 RNAi I. -2h	
	**G**	*control -6h*	4,944	2,26			Unpaired t test with Welch's correction
		UAS-Cnot4 -6h	16,98	5,016	<0,0001	control -6h - Cnot4 RNAi (I.) -6h	
**4.**	**E**	*control -6h*	93,01	6,569			Mann-Whitney test
		UAS-Cnot4 -6h	69,61	6,508	<0,0001	control -6h - Cnot4 RNAi (I.) -6h	
**S1.**	**D**	*control 0h*	21,74	10,3			Kruskal-Wallis + Dunn's multiple comparisons test
		Not1 RNAi 0h	24,29	11,83	>0,9999	control 0h - Not1 RNAi 0h	
		Rcd-1 RNAi 0h	26,12	8,163	0,8038	control 0h - Rcd-1 RNAi 0h	
**S2**	**G**	*control 1h*	85,49	7,889			Kruskal-Wallis + Dunn's multiple comparisons test
		Vps16A RNAi 1h	83,76	9,696	>0,9999	control 1h - Vps16A RNAi 1h	
		Cnot4 RNAi I. 1h	80,82	8,811	>0,9999	control 1h - Cnot4 RNAi (I.) 1h	
		Cnot4 RNAi II. 1h	80,37	8,802	>0,9999	control 1h - Cnot4 RNAi (II.) 1h	
		UAS-Cnot4 1h	15,32	6,592	<0,0001	control 1h - UAS-Cnot4 1h	
	**H**	*Ub+*	10,09	18,61			Kruskal-Wallis + Dunn's multiple comparisons test
		LifeAct+	2,844	1,302	<0,0001	Ub+ - Ub+LifeAct+	
		Ub+LifeAct+	0,6199	0,7202	0,0104	LifeAct+ - Ub+LifeAct+	
